# Isolation and determination of ivermectin in post-mortem and in vivo tissues of dung beetles using a continuous solid phase extraction method followed by LC-ESI^+^-MS/MS

**DOI:** 10.1371/journal.pone.0172202

**Published:** 2017-02-16

**Authors:** Antonio J. Ortiz, Vieyle Cortez, Abdelmonaim Azzouz, José R. Verdú

**Affiliations:** 1 Departamento de Química Inorgánica y Química Orgánica. Universidad de Jaén, EPS de Linares. Linares, Spain; 2 I.U.I. CIBIO, Universidad de Alicante, Alicante, Spain; Indian Institute of Chemical Technology, INDIA

## Abstract

A new analytical method based on solvent extraction, followed by continuous solid-phase extraction (SPE) clean-up using a polymeric sorbent, was demonstrated to be applicable for the detection of ivermectin in complex biological matrices of dung beetles (hemolymph, excreta or dry tissues) using liquid chromatography combined with positive electrospray ionization tandem mass spectrometry (LC/ESI^+^–MS/MS). Using a signal-to-noise ratio of 3:1, the limit of detection (LOD) in the insect matrices at trace levels was 0.01 ng g^–1^ and the limit of quantification (LOQ) was 0.1 ng g^–1^. The proposed method was successfully used to quantitatively determine the levels of ivermectin in the analysis of small samples in in vivo and post mortem samples, demonstrating the usefulness for quantitative analyses that are focused on future pharmacokinetic and bioavailability studies in insects and the establishment of a new protocol to study the impact of ivermectin on non-target arthropods such as dung beetles and other insects that are related with the “dung community”. Because satisfactory precision and accuracy values were obtained in both in vivo matrices, we suggest that the method can be consistently used for quantitative determinations that are focused on future pharmacokinetic and bioavailability studies in insects. Furthermore, this new analytical method was successfully applied to biological samples of dead dung beetles from the field suggesting that the method can be used to establish a new routine analysis of ivermectin residues in insect carcasses that is applied to complement typical mortality tests.

## Introduction

Avermectins are macrocyclic lactone derivatives that are produced during the fermentation of *Streptomyces avermitilis*. Abamectin and ivermectin are the two most widely used compounds in the avermectins group. Ivermectin is an antiparasitic drug used in veterinary applications for the control or eradication of parasites in mammals [1–3]. It has been the mainstay of livestock parasite control since the early 1980s, and over the last decade it has been increasingly used to eradicate several human filarial parasite species [4,5]. Ivermectin has a high molecular weight (MW 860–874), and it has a 16-membered macrocyclic ring that contains disaccharide components. It comprises a mixture of two homologous compounds, 22,23-dihydroavermectin B_1a_ (>80%) and 22,23-dihydroavermectin B_1b_ (<20%) [6,7]. The use of ivermectin is toxic to nervous and growth systems, and it is highly effective at extremely low dose levels (0.2–0.5 mg kg^–1^) against nematode and arthropod species in cattle, sheep, pigs and horses [8–10].

Ivermectin can be available in the form of an injectable solution, which is applied topically; it is highly lipophilic and tends to deposit and accumulate in fat tissue, which could acts as a drug reservoir [11]. The parent H_2_B_1a_ component represents the major fraction of residue in all animal species, and the majority of the dose given to the animal is excreted relatively unaltered in faeces [12]. Numerous field studies have reported strong non-target effects of ivermectin on beneficial arthropods such as the arthropod community that is responsible for decomposing livestock dung, such as dung beetles [10,12,13]. This focus is explained partly by the high persistence of these compounds, partly by their toxicity at extremely low concentrations, and partly by their mode of action; for example, impacting the nervous system of adult and larval insects [12]. Therefore, the widespread use of these antiparasitic drugs could present a potential risk to non-pest insects. A recent study showed that mature dung beetles feeding on dung, even at low concentrations of ivermectin, experience an acute toxicity that alters sensorial and locomotor capacities and consequently, it prevents the performance of normal biological activities such as food detection, interspecific communication, locomotion and interaction with the environment. These results suggest that the decline of several populations of dung beetles that has occurred across Europe could be related to the harmful effects of chemical contamination in dung due to the effects of macrocyclic lactone derivatives [14]. For this reason, the survival time of dung beetles decreases drastically as the amount of ingested ivermectin increases [14]. Consequently, in sites where ivermectin is used as a preventive veterinary medical product, a large number of dead beetles are found in the ground in close proximity to the dung of treated cattle (JRV, personal observations).

Several analytical methods have been developed for the determination or quantification of ivermectin in different matrices, including extraction and clean-up procedures and detection methods (see Table 1 for a review). The methods that are used to analyze ivermectin include thin layer chromatography, immunochemical methods, gas chromatography-mass spectrometry (GC–MS) and liquid chromatography (LC) with UV detection, fluorescence detection (FLD) and mass spectrometry (MS) [15–30]. However, GC–MS and LC–FLD require derivatization of the compounds prior to detection [31]. This additional step is time consuming as well as labor intensive. Recently, a liquid chromatography electrospray ionization (LC–ESI^+^–MS/MS) method was used. The method is direct and the analytes can be analyzed in the same matrix without the need for derivatization [16,23]. Solid phase extraction (SPE) is commonly used as a sample preparation method for several analytical procedures [32,33]. However, in order to use a SPE approach for a broad range of matrix, having different physical and chemical properties, the choice of a single cation-exchange material for the clean-up SPE is limited [34]. A preconcentration step by solid-phase extraction (SPE) and liquid chromatographic separation coupled to mass spectrometry enables very selective and sensitive detection, which makes it a potential alternative to traditional methods. Continuous solid phase extraction is a standard procedure and it has been used for the detection of pharmaceuticals in biological samples [35] or pesticides in drinking water [36].

**Table 1:** Comparison of methods and performance metrics for the determination of ivermectin in different biological and inorganic matrices.

Matrix	Aliquots	Method	Extraction	Clean-up	*r*	*LLOQ*	Recovery (%)	Ref.
Animal (mammal) tissue	2 g	HPLC-FLD	Acetonitrile	SPE alumina	0.999	1.0	70–80	[15]
Animal (mammal) tissue	2.5 g	HPLC-FLD	Acetonitrile	SPE alumina + C18	0.999	2.0	86–97	[16]
Animal (mammal) tissue	5 g	LC-MS; LC-FLD	Acetonitrile + water	LTP	0.99	1.0–2.0	96–100.7	[17]
Animal (mammal) tissue	2.5 g	HPLC/APCI-MS/MS	Acetonitrile	SPE C8	0.997	NA	72.3	[18]
Animal (mammal) tissue	1 g	LC/ESI-MS/MS	Ethanol	LLE	NA	NA	NA	[19]
Animal (mammal) plasma	500 μl	LC/ESI-MS/MS	Acetonitrile	SPE C18	0.9989	1.0	NA	[20]
Human plasma	200 μl	HPLC-FLD	Acetonitrile + water	SPE Oasis	0.9992	0.2	86	[21]
Plant-based matrices	5 g	LC/ESI-MS	Acetonitrile	SPE alumina	0.999	0.53	89–102	[22]
Dairy products	5 ml	LC/ESI-MS	Carrez's reagent + methanol	on-line SPE	0.998	2.11	82–88	[23]
Dairy products	0.5–1.0 g	HPLC-FLD	Acetonitrile + ethyl acetate + water	LLE	0.998	0.16	84.6–106.5	[24]
Dairy products	5 g	LC/ESI-MS/MS	Acetonitrile + methanol	LLE	0.999	0.2	92–100	[25]
Edible oils	2.5 g	LC-MS/MS	Acetonitrile	LTP	0.99	1.1–0.3	71.1–119.3	[26]
Foodstuffs	2.5 g	HPLC-FLD	Acetonitrile	LLE	0.99	NA	67.9–88.9	[27]
Water, sediment and soil	1–5 g	HPLC/APCI-MS/MS	PLE methanol + water	SPE alox-N; C18; Oasis	0.99	0.5–2.5	73–81	[28]
Reindeer feces	1 g	HPLC-FLD	Acetone + isooctane	SPE C18	NA	NA	95–116	[29]
Cattle feces	15 ml	HPLC-FLD	Acetone + isooctane	SPE C18	NA	2	84	[30]

Legend: (HPLC-FLD) High-performance liquid chromatography with fluorescence detection; (LC-MS) Liquid chromatography–mass spectrometry; (LC-FLD) Liquid chromatography with fluorescence detection; (HPLC/APCI-MS/MS) High-performance liquid chromatography–atmospheric pressure chemical ionization–tandem mass spectrometry; (LC/ESI-MS/MS) liquid chromatography-electrospray ionization-tandem mass spectrometry; (PLE) pressurized liquid extraction; (SPE) solid-phase extraction; (LTP) temperature purification; (LLE) liquid–liquid extraction; (*r*) linearity, the correlation coefficient; (*LLOQ*) lower limit of quantification, expressed in ng ml^-1^; (NA) not available.

The present study is focused on the development of a simple detection method without derivatization and further clean-up for ivermectin in insect-derived biological samples using continuous solid phase extraction and LC-ESI-MS. The aim of the current work was to develop a reliable and sensitive method for the routine analysis of ivermectin from various matrices (hemolymph, insect excretes and dry insect carcasses) and there are two different goals. The first goal is to quantify the ivermectin concentration from in vivo samples such as hemolymph or excretes from living beetles to establish future studies about the pharmacokinetics and bioavailability of ivermectin in dung beetles. For this purpose, the model species was *Scarabaeus cicatricosus* Lucas, 1846, which was selected due to its local abundance and functional relevance in Mediterranean ecosystems [14]. The second goal is to describe a simple extraction procedure that is applicable to dry carcasses of insects to establish a new protocol for necropsy in support of a diagnosis of toxicity of ivermectin in dung beetles that die in the field. In this case, we selected *Scarabaeus cicatricosus* and *S*. *sacer* Linnaeus, 1758, which was selected due to its high frequency of mortality that is observed in the field (JRV personal observations). This new method was applied for the detection of ivermectin in 36 post-mortem samples of *Scarabaeus cicatricosus* and *S*. *sacer* carcasses that were collected from a site where ivermectin is used as a veterinary pharmaceutical treatment for cattle.

## Materials and methods

### Standards and chemicals

The Abamectin (98.7%) and ivermectin (90% B_1a_; 5% B_1b_) standards were provided by Sigma Aldrich (St. Louis, USA), and they were stored at –20°C. Abamectin, a precursor to ivermectin, differs from ivermectin in that it has a double-bond at the C_22–23_ position, and it was used as an internal standard (IS). Acetonitrile and methanol (HPLC grade) were supplied by Panreac (Barcelona, Spain). Oasis-HLB^®^ sorbent was obtained from Waters Corporation (Milford, Massachusetts, USA). Ammonium formate and formic acid (analytical reagent grade) were purchased from Merck (Darmstadt, Germany). Millex-LG filter units (hydrophilic, PTFE, pore size = 0.20 μm, diameter = 25 mm, filtration area = 3.9 cm^2^) were obtained from Millipore Ibérica. Water was purified by passage through a Milli-Q system (Millipore Ibérica, Madrid, Spain).

To prepare the stock solutions, approximately 10.0 mg ± 0.01 mg of ivermectin and abamectin reference standards were accurately weighed into individual 100 ml volumetric flasks and dissolved using methanol, to prepare two standard solutions of 100 μg ml^–1^ for ivermectin and abamectin. Then, the appropriate aliquots were obtained and further diluted with methanol to give a series of working solutions with a concentration ranging from 0.1 to 1000 ng ml^–1^. The IS working solution concentration was 5 ng ml^–1^. All the solutions were stored in a freezer at –20°C.

### Dung beetles and sampling design

Dung beetles (*Scarabaeus cicatricosus* and *S*. *sacer*) were collected from the Doñana Biological Reserve (DBR-ICTS), an ivermectin-free site within the Doñana National Park (Huelva), in Southern Spain during the summer (July 2014).

For ivermectin detection in the hemolymph and excretes of beetles, only individuals of the most abundant species, *Scarabaeus cicatricosus*, were collected. Individuals were maintained in plastic containers (60 × 40 × 40 cm) with moist sterile vermiculite as the substrate at 28–30°C in a climate-controlled chamber (a temperature similar to the optimal one experienced in the field). For this bioassay, two concentrations of fresh cow dung-ivermectin, 1.0 (ivermectin T1) and 100.0 (ivermectin T100) μg kg^–1^, and an untreated control were used. Previously, dung ivermectin concentrations were achieved by dissolving ivermectin in absolute ethanol (Sigma-Aldrich Co.), and 2 ml aliquots of the two selected concentrations were added to 2 kg portions of fresh cow dung and mixed for 20 min using a kitchen mixer. For the untreated control, absolute ethanol (2 ml) was applied to the same quantity of dung. Residual ethanol was removed by evaporation during the 6 hours before transferring the dung treatments to the individual experimental units. To identify ivermectin in the hemolymph and excretes, 10 individuals were fed 4 ml of both dung treatments. The beetles in the control group (*N* = 10) were treated identically to the treatment groups but they were fed the free ivermectin dung. After three days, the dung that was not consumed was removed, and a new portion of the corresponding dung treatment was added. Twenty days post-treatment, all animals were allocated to collecting hemolymph. A one-way ANOVA test was used to analyze possible differences in the concentration of ivermectin between the T1 and T100 treatments.

For post-mortem analysis, the dung beetles carcasses (from both *Scarabaeus* species) that were observed in the field were collected in July and October 2014 from Los Sotos, a site within the Doñana National Park where ivermectin is used as a veterinary pharmaceutical treatment for cattle and the dead dung beetles in close proximity to dung have been observed periodically. The beetle carcasses were transported individually in plastic vials, identified and stored at −20°C until analysis.

This work conforms to the Spanish legal requirements including those relating to conservation and welfare. Moreover, beetle collection was conducted with relevant permission related to collection and field study.

### Sample preparation and extraction

**Fig 1:**
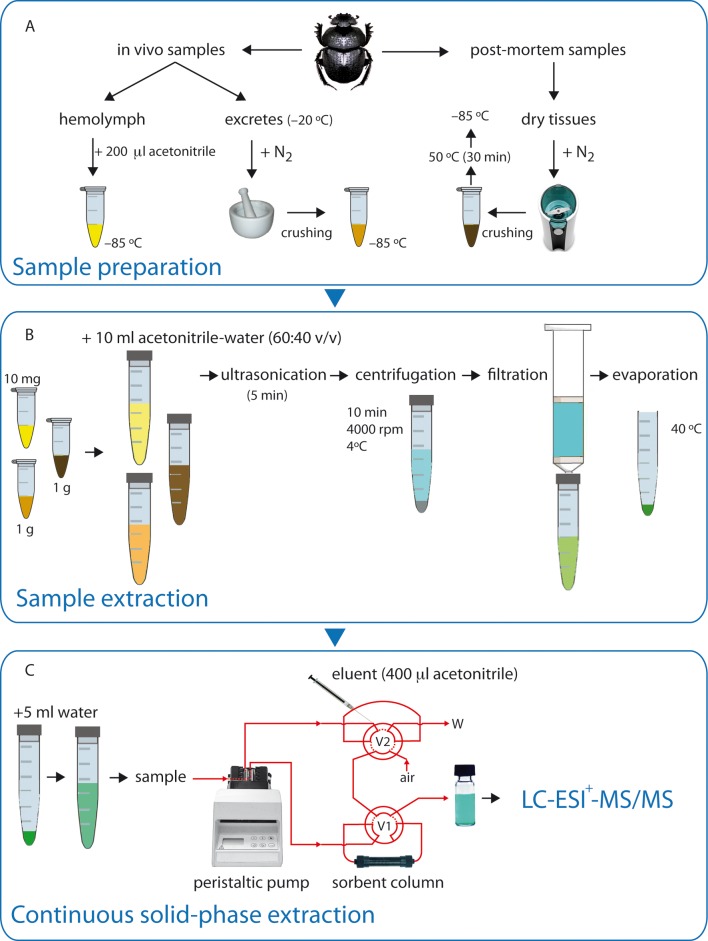
Schematic overview of methods used to determine ivermectin levels in different insect matrices. (A) Sample preparation in hemolymph and excretes (in vivo samples) and dry carcasses of dung beetles (post mortem samples); (B) Sample extraction procedure, and (C) Continuous solid-phase extraction (V1: valve 1; V2: valve 2; W: waste).

The sample preparation technique varied based on the sample nature. A flow diagram for the protocol is presented in Fig 1.

For in vivo samples, hemolymph was collected by puncturing the cuticle on the dorsal side of the abdomen and gently squeezing the insect. The exuded hemolymph was collected with a sterile syringe. The amount of collected hemolymph equivalent to 3 individuals (∼10 μl) was placed into a glass vial with 200 μl of acetonitrile, protected from light and stored at −85°C in an ultrafreezer (SANYO Electric Co. Ltd, Japan). The beetles were frozen immediately after collecting their hemolymph. Excreta were obtained from treated and untreated beetles every 3 days. Then, the excreta samples were placed individually into plastic vials and stored at −20°C until analysis. The sample excretes from each treatment were placed in a porcelain mortar in which liquid nitrogen was poured, and after crushing, the sample was stored at −85°C in an ultrafreezer (SANYO Electric Co. Ltd, Japan).

For insect carcasses (see post-mortem samples in Fig 1), the *Scarabaeus sacer* samples were prepared using only a carcass/sample; however, due to the lower mass of *S*. *cicatricosus*, in this case two carcasses were required for each sample. Each sample was placed in a mixer (Taurus 50N, Spain) container in which liquid nitrogen was poured while it was crushed, and then they were placed in an oven at 50°C for 30 minutes. Approximately 1.0 ± 0.01 g of each sample equilibrated at room temperature was placed in a 15 ml polypropylene centrifuge tube and extracted with 4 ml of water and 6 ml of acetonitrile (see sample extraction in Fig 1B). Next, the mixture was sonicated for 5 min in an ultrasonic bath (JP Selecta, Barcelona, Spain), and then the tubes were shaken for 1 min in a vortex (REAX Control, Heidolph, Kelheim, Germany). The acetonitrile phase was separated by centrifugation on a Centrofriger BL-II apparatus (JP Selecta, Barcelona, Spain) at 1914 g for 10 min (4°C). Immediately following centrifugation, the supernatant layer was transferred to a clean tube where it was evaporated to dryness at 40°C under a stream of ultrahigh-purity N_2_.

Afterwards, the pre-concentration and clean-up of samples were performed using continuous solid phase extraction (SPE) (Fig 1C). The continuous SPE technique used for the pretreatment for samples was based on the methodology described by Azzouz et al. [34]. The continuous solid-phase extraction (SPE) manifold used was assembled from a Gilson Minipuls-3 peristaltic pump (Villiers-le-Bel, France) fitted with polyvinyl chloride (PVC) pumping tubes, two Rheodyne 5041 injection valves (Cotati, CA, USA) and PTFE (3 mm I.D.) and laboratory-prepared columns of variable length packed with each sorbent material. The Oasis-HLB^®^ sorbent columns were conditioned by passing 1 ml of acetonitrile and 1 ml of purified water. Under these conditions, the column remained active for more than 50 samples. In the preconcentration step, 5 ml of the pretreated sample was filtered to prevent the suspended particles from reaching the continuous unit, and they were passed at 4 ml min^−1^ through the sorbent column (80 mg Oasis-HLB^®^). Ivermectin was adsorbed and the sample matrix was sent to waste. The analytes were eluted with 400 μl of acetonitrile in a glass vial. Then, this solution was filtered through a Millipore PTFE 0.20 μm (Millex^®^ Syringe Filter Units, Sterile, 25/50/62 mm, Hydrophobic PTFE) filter and stored −20°C until analysis in the dark.

### LC/ESI^+^–MS/MS analysis

The LC/ESI–MS/MS system consisted of an Agilent HPLC system Type 1100 linked to an Ion Trap MS analyzer (Esquire 6000 de Bruker Daltonics) equipped with an electrospray ion source (ESI). The system was controlled with the software packages Agilent ChemStation (version A.06.01, Agilent Technologies) and Bruker Daltonics esquire control (version 6.08, Bruker Daltonics, Bremen, Germany). The data were processed with Data Analysis software (version 3.2, Bruker Daltonics).

Chromatography was performed on a C_18_ Kinetex column (75 mm × 3.0 mm, 3.0 μm, Phenomenex, USA) that was operated at 40°C. The mobile phase solvent A was a solution of 0.1% (v/v) formic acid in water and solvent B was 0.1% (v/v) formic acid in acetonitrile. The column flow rate was 0.25 ml min^–1^, the injection volume was 1 μl and the gradient elution timetable was as follows: 0–10 min 50% A-50% B; 10–15 min, 100% B; 15–20 min 50%A-50%B.

One-third of the column eluent was sent via ESI to the Ion Trap detector. For MS/MS detection, electrospray was operated in the positive ion mode, and the ionization source parameters were as follows: capillary voltage, 4.0 kV; octopole RF amplitude, 144.3 Vpp; cone voltage 40 V; nebulizing gas pressure, 40 psi; drying gas flow rate, 9 l min^–1^; capillary exit voltage, 300 V; and desolvation temperature, 350°C. Instrument tuning was performed for ivermectin and abamectin by direct infusion of a 1 μg ml^–1^ solution. The optimum collision energy in the MS/MS mode for the protonated sodium adducts of the molecular ion for ivermectin (*m/z* 897.4) and abamectin (*m/z* 895.4) was 40 eV. For quantitative purposes, the instrument was operated in the multiple reaction monitoring (MRM) mode, scanning from 50 to 2000 *m/z*.

### Method validation

The proposed method for the quantitative determination of ivermectin in post mortem and in vivo tissues was validated by a set of established parameters. The linearity of the method was evaluated using fortified ivermectin-free dung beetles. Six evenly distributed calibration standards of ivermectin B_1a_ concentrations were used: 0, 0.1, 0.5, 5.0, 7.0 and 10.0 ng g^–1^. A fixed concentration of the abamectin (5 ng g^–1^) internal standard (IS) was added to the samples immediately prior to the analysis. The peak area ratios between ivermectin B_1a_ and the IS was plotted against the concentration ratios and a linear regression was performed. The acceptance criterion for the correlation coefficient (*r*) was *r* ≥ 0.98.

The limit of detection (LOD) was determined by considering a signal to noise (S/N) ratio of 3:1 and the limit of quantification (LOQ) was determined from the lowest concentration of ivermectin (in spiked ivermectin free-insects), which followed the criteria of a signal-to-noise ratio (S/N) of 10:1. Matrix-matched standard calibration curves were generated by preparing insect carcasses as described above and then mixing them with standard solutions containing ivermectin at a concentration ranging from 10 ng g^–1^ to 0.1 ng g^–1^. LOQ was established as the lowest point of the calibration curve.

The recovery of ivermectin was assessed by analyzing ivermectin-free insects that were spiked at two concentrations 0.6 and 6.0 ng g^–1^ in triplicate. The recoveries were determined by extrapolating the analyte concentrations from the calibration curves.

## Results and discussion

A new analytical method was developed based on continuous solid-phase extraction to detect ivermectin in post-mortem and in vivo tissues of dung beetles using LC-ESI-MS/MS. The extraction step and clean up using an acetonitrile-water mix resulted in a clean extract. The method described in this paper requires a short extraction time and small volumes of solvents using small quantities of sample.

### Optimization of extraction procedure and method validation

Various solvents, including acetonitrile [17,22] or ethanol [21] have been extensively used for extracting ivermectin residues from biological samples. The selection of the solvent therefore depends on not only the target compound, but also the matrix and in the present study, after optimization of the extraction conditions, the optimal extraction solvent consisted of acetonitrile and distilled water at a ratio of 3:2 (v:v). Extraction using an acetonitrile-water (60:40, v/v) mixture was highly efficient, with minor interference from impurities, and that mixture was selected as the extraction solvent in the following work. The mixture was also used as the solvent for the extraction of the spiked insect tissue samples (6.0 and 0.6 ng g^−1^ fortification level). In order to avoid the problems associated to IMV contamination from previous samples, it was checked by analyzing blank samples of 4 ml of ultrapure water every 3 samples analysis. No carry-over was observed from the sorbent material during the tests in the method.

The results of the method validation are summarized in Table 2. All the validation parameters indicated that the protocol to detect ivermectin in dung beetle tissues (for living and dead beetles) and hemolymph was highly precise, sensible and reproducible (see Table 1 for a review of the different analytical procedures). The samples that were spiked at two different concentration levels (0.6 and 6 ng g^–1^) exhibited a recovery percentage range (mean ± s.d.) from 91.1 ± 11.6% to 103.2 ± 6.4%, respectively. These results indicate that after the continuous SPE extraction, ivermectin can be extracted completely from beetle tissue samples and that the macrolides lactone loss is negligible during the whole analytical procedure. Furthermore, the concentration detected by the instrument with a 3:1 S/N ratio is interpreted as the ivermectin limit of detection, which is estimated to be 0.01 ng g^–1^. The concentration detected by the instrument with a 10:1 S/N ratio is interpreted as the limit of quantification, and it is estimated to be 0.1 ng g^−1^. This value is lower than the result in many of the previous studies (Table 1). Finally, the calibration standards were run in triplicate and the calibration curve showed good linearity in the range of 0.1 ng ml^–1^ to 10 ng ml^–1^ (*r =* 0.988). Six calibration standards distributed evenly over the concentration range of interest were analyzed and were run in triplicate.

**Table 2:** Validation results for the determination of ivermectin B_1a_ in dung beetle samples by LC-ESI^+^-MS/MS.

Parameters		Validation results
Calibration curve	Concentration range	Correlation coefficient (*r*)
	0.1 to 10 ng g^–1^	0.988
Lower limits	Concentration range	
Lower limit of detection (*LLOD*)	0.1 to 10 ng g^–1^	0.01 ng g^–1^
Lower limit of quantification (*LLOQ*)	0.1 to 10 ng g^–1^	0.1 ng g^–1^
Recovery of IVM added to carcass tissue	Spiked with	Recovery ± SD (%)
	0.6 ng g^–1^	91.1±11.6
	6 ng g^–1^	103.2±6.4
Specificity	No interference of endogenous compounds	

### LC-ESI-MS/MS optimization

The best LC-MS/MS ionization conditions were achieved using direct infusion electrospray solvent (acetonitrile/water) ammonium formate buffer operated in negative and positive mode. However, the positive ion signals were considerably larger than the negative ion signals, and the signals were detectable at very low concentrations of ivermectin and internal standard of abamectin (IS).

**Fig 2:**
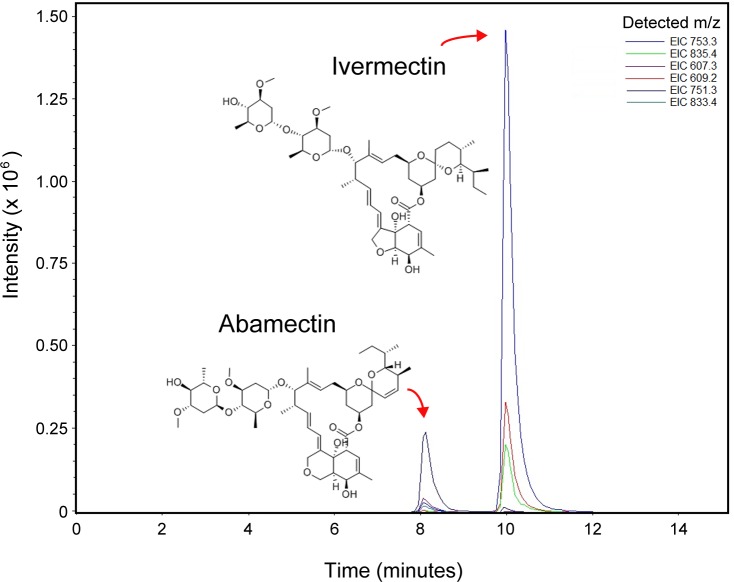
LC-ESI^+^-MS/MS total ion chromatogram: abamectin (Retention time: 8.2 min) and ivermectin B_1a_ (Retention time: 10.1 min).

**Fig 3:**
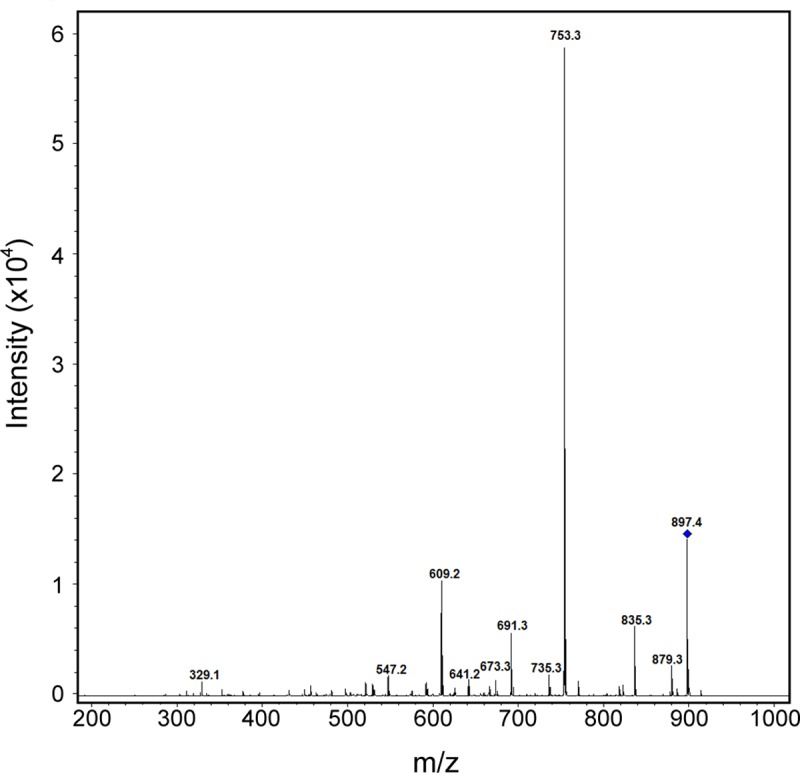
ESI tandem mass spectrum of ivermectin B_1a_ showing a peak at *m/z* 897.4 corresponding to [B_1a_+Na]^+^ ivermectin.

The chromatographic separation is shown in Fig 2. Under the chromatographic conditions mentioned above in the materials and methods section, the retention times for ivermectin and IS were 10.1 and 8.2 min, respectively. The total run time per single injection was less than 11 min. Fig 3 shows the full-scan MS/MS spectrum of ivermectin obtained in the positive ion mode and using the tune parameters mentioned above. In ESI positive ion MS mode, the mass spectra of ivermectin showed a principal peak at *m/z* 897.4 from [B_1a_+Na]^+^ rather than the expected *m/z* 875 [B_1a_+H]^+^, and three main daughter ions at *m/z* = 753.3, 835.3 and 609.4 showed adducts with sodium as the additive peak, which has also been described in other studies [19,20]. For abamectin (IS) the *m/z* 895.7 ion was the most abundant and three additional daughter ions were detected at *m/z* 833.4, 751.3 and 607.3 which match the reported data in the positive ionization mode [19,20,22,23]. Those additional daughter ions were used for the complete identification and quantification of ivermectin. For quantitative purposes, the instrument was operated in the multiple reaction monitoring (MRM) mode using the daughter ions at *m/z* 753.3, 835.4 and 607.3 for ivermectin and *m/z* 751.3, 833.4 and 605.2 for abamectin.

### Ivermectin determination in vivo samples

Ivermectin was detected in all analyzed matrices: hemolymph extracted samples and dung beetle excreta.

**Fig 4:**
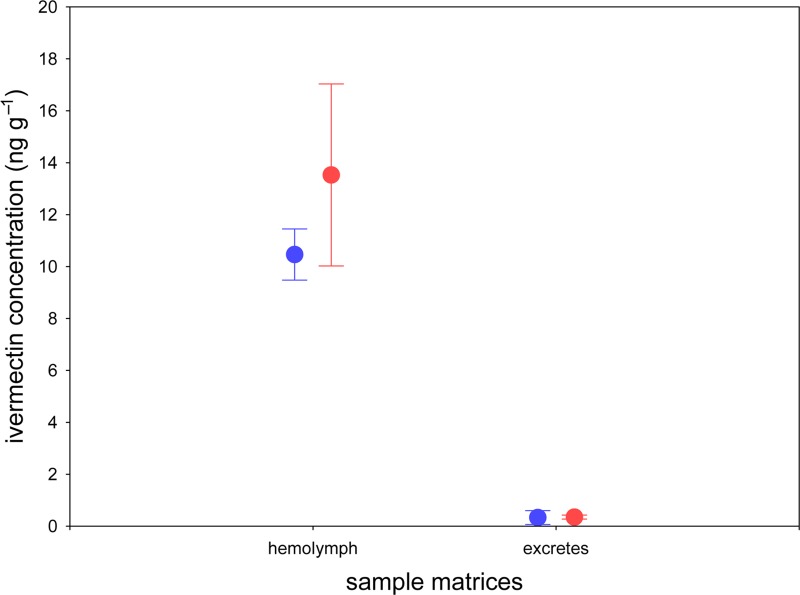
Ivermectin determination in hemolymph and excretes of *Scarabaeus cicatricosus*. Mean (±s.d) data showing ivermectin concentrations in T1 and T100 treatments.

For hemolymph samples, ivermectin was detected in both beetle groups that were treated with the selected standard doses (T1 and T100) of ivermectin (Fig 4). High quantities of ivermectin were detected in both treatments (T1: 10.47 ± 0.40 ng g^−1^; T100: 13.53 ± 2.20 ng g^−1^). We observed a positive relation between ivermectin concentration in the treatment and the quantity of ivermectin detected; however, no significant differences between both treatments were found (*F* = 5.41, *P* = 0.06). No ivermectin was detected in any of the hemolymph samples from the control group (*N* = 10).

For excreta samples, ivermectin was determined in both beetle groups that were treated with the selected standard doses (T1 and T100) of ivermectin (Fig 3). Compared with the hemolymph samples, smaller quantities of ivermectin were determined in both treatments (T1: 0.33 ± 0.11 ng g^−1^; T100: 0.35 ± 0.03 ng g^−1^). No significant differences between both treatments were found (*F* = 0.08, *P* = 0.795).

Despite the 100-fold higher dose, the concentration in hemolymph and excreta from the T100 samples was not significantly higher than the 10-fold lower concentration (T1). Furthermore, the ivermectin concentration in hemolymph was approximately 10-50-fold higher than in the respective excreta extract. This interesting result could suggest the potential bioaccumulation of ivermectin in dung beetles as occurs in other invertebrate species such as *Lumbriculus variegatus* [37]. The pharmacokinetic properties of ivermectin affect the function of each species in which the compound is studied [38]. Surprisingly, no data exist about the pharmacokinetics of ivermectin in dung beetles, which explains why this new analytical method could be the basis for future studies about the pharmacokinetics and bioavailability of this drug in different dung beetle species and other affected insects. Because the available information about the metabolites and products of ivermectin in insects is not sufficient, further research is needed to determine transformation products of ivermectin, such as the monosaccharide (22,23-dihydroavermectin B1 monosaccharide) and the aglycon of ivermectin (22,23-dihydroavermectin B1 aglycon) [39].

### Ivermectin determination in post mortem samples

**Fig 5:**
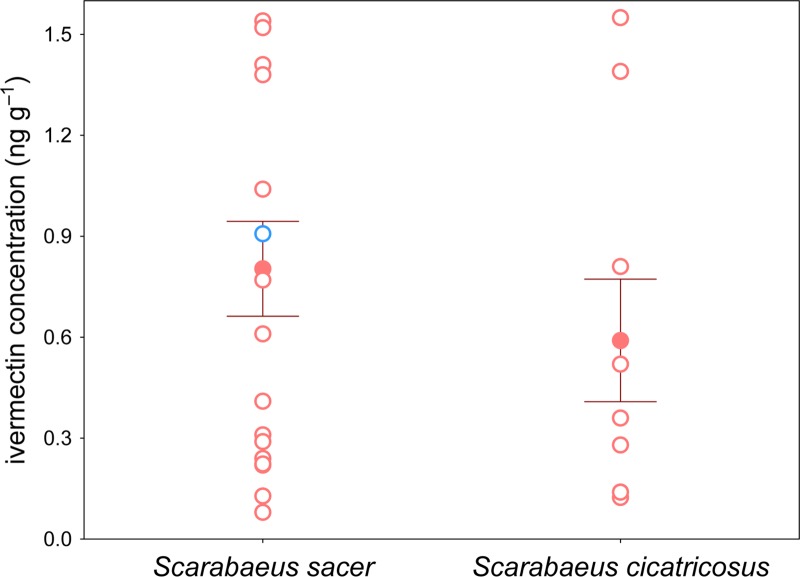
Ivermectin determination in carcasses of *Scarabaeus sacer* and *S*. *cicatricosus*. Mean (±s.d) data showing ivermectin concentrations in both species (red filled dots). Unfilled red dots correspond to individual samples; unfilled blue dot corresponds to individual of *S*. *sacer* in which the time of death exceeded three months. In all cases, samples were directly collected in the field (partially dehydrated by field conditions).

A total of 36 dead *Scarabaeus cicatricosus* (*N* = 18) and *S*. *sacer* (*N* = 18) beetles were found in the field. In all cases the carcasses were intact without any indications of death caused by crushing or predatory attack. The time of death was not determined with precision but in all cases it was more than one week. The time of death surpassed three months in only one case (for *S*. *sacer*). In all cases, ivermectin was detected in dead beetles with the exception of only one *S*. *cicatricosus*. The concentration of ivermectin in carcass tissues was (mean ± SD, in ng g^−1^) 0.59 ± 0.55 and 0.80 ± 0.60 for *Scarabaeus cicatricosus* and *Scarabaeus sacer*, respectively (Fig 5). This result suggests that the new analytical method is a useful tool to establish a new protocol to study the impact of ivermectin on non-target arthropods such as dung beetles and other insects that are related with the “dung community”. According to our results, ivermectin residues remain in the insect carcasses, which is likely due to the function of the insect cuticle in preventing the passage of ultraviolet rays that are capable of ivermectin transformation. As mentioned above, a *S*. *sacer* carcass was found that had been dead for three months, and ivermectin was detected in the carcass (Fig 5). Thus, the high resistance of the ivermectin molecule together with the physiochemical nature of the insect cuticle enables drug detection in the carcasses of dung beetles for a long period of time.

### Conclusions

To the best of our knowledge, the proposed method is the first published qualitative and quantitative method for the determination of ivermectin B_1a_ in insect tissues using LC-ESI^+^-MS/MS. A clear drawback of living organism’s sample-preparation procedures is the occurrence of abundant matrix effects, which compromise quantification limits, analysis time and therefore analysis costs. The developed method allows the detection and quantification of ivermectin in very complex biological matrices that are present at trace levels with high extraction recoveries, accuracy and sensitivity using the continuous solid phase extraction procedure. SPE in cartridge is frequently used as a clean-up technique in the simultaneous analysis of IVM in matrix of animal origin, but selective wash steps and a selective elution can be used effectively to separate the target compounds from matrix interferences [20,21,22,40]. Because in our study, the chemical properties of the matrix (post morten dung beetle tissues hemolymph and excreta) are very diverse, the options for clean up in SPE cartridges are limited. Although a simplified extraction procedure was used, no interferences were observed from the matrix components during the determination of ivermectin residues. The combination of the selected fast extraction technique with the use of continous SPE and LC-MS/MS permits the performance of the analysis of IVM residues more simple, cost-effective (the column remains active for months) and less time-consuming than traditional methods. Because satisfactory precision and accuracy values were obtained in both in vivo matrices, we suggest that the method can be consistently used for quantitative determinations that are focused on future pharmacokinetic and bioavailability studies in insects. Furthermore, this new analytical method was successfully applied to biological samples of dead dung beetles from the field (post-mortem analysis). Given that guidelines established by the International Cooperation on Harmonisation of Technical Requirements for Registration of Veterinary Medicinal Products (VICH; http://www.vichsec.org/) require an environmental risk assessment when animal excreted residues such as ivermectin are considered that adversely affect non-target organisms [10], we suggest that the method can be used to establish a new routine analysis of ivermectin residues in insect carcasses that is applied to complement typical mortality tests.
